# Effect of different carbon sources on the biological phosphorus removal by a sequencing batch reactor using pressurized pure oxygen

**DOI:** 10.1080/13102818.2014.924200

**Published:** 2014-07-10

**Authors:** Jie Wei, Tsuyoshi Imai, Takaya Higuchi, Novi Arfarita, Koichi Yamamoto, Masahiko Sekine, Ariyo Kanno

**Affiliations:** ^a^ Graduate School of Science and Engineering, Division of Environmental Science and Engineering, Yamaguchi University, Yamaguchi, Japan; ^b^ Faculty of Agrotechnology, Malang Islamic University, Malang, Indonesia; ^c^ Systems Design and Engineering, Graduate School of Science and Engineering, Yamaguchi University, Yamaguchi, Japan

**Keywords:** pressurized pure oxygen sequencing batch reactor (POSBR), polyphosphate-accumulating organisms (PAOs), carbon source, biological phosphorus removal

## Abstract

The effect of different carbon source on the efficiency of enhanced biological phosphorus removal (EBPR) from synthetic wastewater with acetate and two ratios of acetate/starch as a carbon source was investigated. Three pressurized pure oxygen sequencing batch reactor (POSBR) experiments were operated. The reactors (POSBR1, POSBR2 and POSBR3) were developed and studied at different carbon source ratios of 100% acetate, 75% acetate plus 25% starch and 50% acetate plus 50% starch, respectively. The results showed that POSBR1 had a higher phosphate release-to-uptake ratio and, respectively, in a much higher phosphorus removal efficiency (93.8%) than POSBR2 (84.7%) and POSBR3 (77.3%) within 30 days of operation. This indicated that the phosphorus removal efficiency decreased the higher the starch concentration was. It was also found that POSBR1 produced more polyhydroxyalkanoates (PHAs) than the other reactors. Based on the effect of the carbon source on the PHA concentration and consumption, the conditions of POSBR1 were favourable for the growth of polyphosphate-accumulating organisms and therefore, beneficial for the biological phosphorus removal process.

## Introduction

In the last 30 years, nutrient enrichment has been recognized as a major threat for the condition of marine ecosystems and resources. Among the large array of nutrients in wastewater, phosphorus compounds are considered a major cause of eutrophication. The most effective and most commonly used method for phosphorus removal is biological treatment. The primary characteristic of enhanced biological phosphorus removal (EBPR) systems is the alternating condition of anaerobic and aerobic environments to stimulate the growth of phosphorus-accumulating organisms (PAOs), which are capable of storing volatile fatty acids (VFAs) as internal storage compounds, polyhydroxyalkanoates (PHAs), under anaerobic conditions.[1] The energy required for this assimilation comes from intracellular degradation of polyphosphate, which is then released into the mixed liquor. Under subsequent aerobic conditions where external carbon substrates are absent, bacteria use the stored PHAs to produce energy for cell growth and maintenance as well as uptake of phosphate from the liquid to build up polyphosphate.[2] The biochemical mechanisms of EBPR have been studied in detail by many authors.[1–5] In this study, a sequencing batch reactor (SBR) process was carried out using pressurized pure oxygen to treat wastewater. Pure oxygen aeration technology gained rapid development with many advantages, such as high oxygen transfer ratio, low-energy consumption, increased efficiency without large facilities, high efficiency of sewage treatment, resistance to shock loading, less excess sludge, etc.[6,7]

One of the key points in EBPR systems is the carbon source used by PAOs. Modelling efforts to explain the EBPR mechanism have mostly used VFAs as a fermented substrate, such as acetate and propionate,[8–10] and rarely other carbon sources. Oehmen et al. [11] suggested that propionate as a carbon source could be more beneficial for obtaining the desirable level of PAOs in the sludge, but with acetate as a carbon source good phosphate removal efficiency and the highest PHAs production would be attained as well.[12,13] Pijuan et al. [13] also concluded that propionate as a carbon source could be used to provide selective advantage for PAOs. Rustrian et al. [14] observed that acetate and butyrate are equally good carbon sources for phosphorus removal.

Other organic substrates, apart from VFAs, that can also be utilized anaerobically by PAO-enriched sludge include various carboxylic acids, sugars and amino acids.[15] Puig et al. [16] reported that the treatment performance using ethanol as carbon source was close to that with propionate and slightly lower than that with acetate. Several studies have demonstrated that good phosphorus removal efficiency can be achieved if glucose, a mixture of glucose and peptone or a mixture of glucose and acetate is supplied as the carbon source.[12,17,18] In contrast, starch has not been widely used in EBPR processes and the phosphate removal was observed to be maximum 66% in synthetic phosphate wastewater with starch as the carbon source.[19]

When the amount of biodegradable organic substrate is limiting, extra substrate can be added. The choice of the carbon source depends on the economics of the process as well as on the phosphorus removal effectively. For this reason, the present study investigated the potential of using starch as the carbon source for biological phosphorus removal via SBR processes using pressurized pure oxygen with different acetate/starch ratios. Three experiments were conducted, and the factors affecting the removal rate were discussed. We based this research on laboratory phosphorus removal systems with different aeration times described previously.[20]

## Materials and methods

### Batch-scale reactor

The experiments were carried out in a laboratory-scale pressurized oxygen aeration sequencing batch reactor (POSBR) with a working volume of 14 L previously described.[20] Briefly, the microorganism inoculum (aerobic sludge) was obtained from the Eastern Municipal Wastewater Treatment Plant in Ube City, Yamaguchi, Japan. Three reactors were operated in three cycles of 8 h per day. Each cycle consisted of 3 h anaerobic period, 2.5 h aerobic period, 2.5 h sludge setting and supernatant replacement. The reactor was operated at 21 °C ± 1 °C, and pH was not controlled but was kept in the alkaline range during the whole operation period.[21] The reactors were continuously stirred but anaerobic or aerobic conditions were maintained by gassing with nitrogen during the anaerobic period and using oxygen during the aerobic period. The entire installation is sealed except for a small inlet with a spanner valve on top of the reactor. The nitrogen and oxygen gases circulate from the upper part of the reactor to the pump, while the gas strikes the aerator at the bottom of the device, resulting in the production of many bubbles. For a schematic diagram see.[20]

### Synthetic media

After each cycle was completed, the upper supernatant liquid (7 L) was discharged and an additional 7 L concentrated fresh medium was added to the reactor. The components of the synthetic wastewater were as follows: NH_4_Cl (100.8 mg/L), peptone (5 mg/L), NaH_2_PO_4_·2H_2_O (75.5 mg/L), KCl (72 mg/L), NaHCO_3_ (225 mg/L), MgSO_4_·7H_2_O (180 mg/L), CaCl_2_·H_2_O (14 mg/L), yeast extract (5 mg/L) and mineral salts solution 0.3 mL/L.[22] The mineral salts solution was composed of FeCl_3_·6H_2_O (1500 mg/L), H_3_BO_3_ (150 mg/L), CuSO_4_·5H_2_O (30 mg/L), KI (180 mg/L), MnCl_2_·4H_2_O (120 mg/L), Na_2_MoO_4_·2H_2_O (60 mg/L), ZnSO_4_·7H_2_O (120 mg/L), CoCl_2_·6H_2_O (150 mg/L) and EDTA (10 g/L).[23] At loading, the chemical oxygen demand (COD) and phosphate-phosphorus (PO_4_-P) amount in the reactor were 400 mg/L and 15 mg/L, respectively. Three POSBRs were conducted with different carbon sources as given in Table 1: POSBR1 (100% acetate), POSBR2 (75% acetate + 25% starch) and POSBR3 (50% acetate + 50% starch). Sampling was performed at the beginning of the anaerobic period and at the end of the anaerobic and aerobic phases to analyse the variation of phosphate concentration, respectively. During the last cycle of each POSBR, about 10 mL of the wastewater was extracted every 0.5 h to investigate the treatment performance within the operation.

**Table 1.** Different carbon sources in POSBRs.

	POSBR1	POSBR2	POSBR3
CH_3_COONa (mg/L)	586	440	293
Soluble starch (mg/L)	–	93	186

### Analytical methodology

Liquid samples were taken from each POSBR and analysed for VFAs, PO_4_-P, PHAs, oxidation–reduction potential (ORP), mixed liquor suspended solid (MLSS) and mixed liquor volatile suspended solid (MLVSS). VFA was determined by gas chromatography GC-8APF with flame ionization detector (FID), Shimadzu, Japan. MLSS and MLVSS were quantified according to the standard methods.[24] All the samples were filtered through a 0.45 μm membrane filter. The concentration of PO_4_-P was determined colourimetrically by using Toluidine Blue. Filtered sludge samples were completely lyophilized to measure the content of PHA. Extraction and estimation of PHA were performed according to the modified method of Shi et al.[25] The pH and ORP of the samples were measured using a HORIBA pH meter, D-51 and D-52 (Horiba Co. Ltd., Tokyo, Japan), respectively. The DO concentrations were estimated using a HACH LDO^TM^ HQ_10_ analyser (Hach Corp., CO, USA).

## Results and discussion

### PO_4_-P removal performance of POSBRs

**Figure 1.**
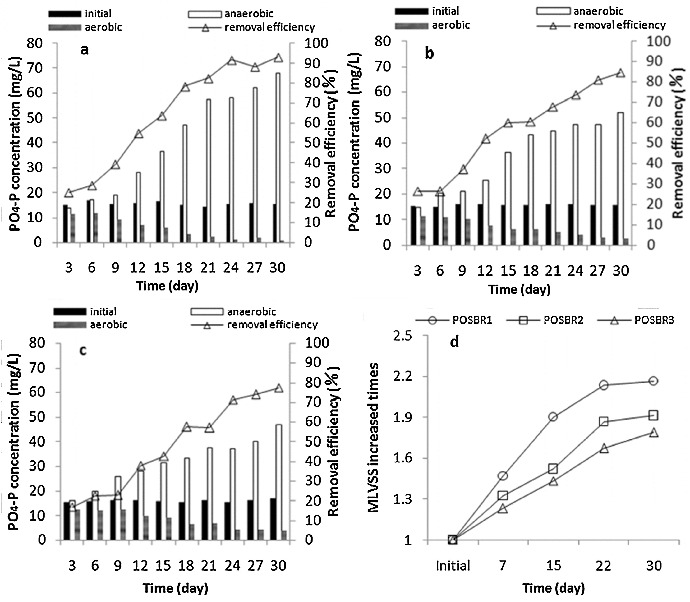
PO_4_-P concentration and removal efficiency of three POSBRs: POSBR1 (a),[20] POSBR2 (b) and POSBR3 (c); number of times that the MLVSS concentration increased compared to the initial value in three POSBRs (d).

The experiments involved three POSBR systems operated at steady state and differing only in the organic carbon source added at the beginning of the anaerobic phase: one was fed with acetate and the others with different combinations of acetate/starch ratios, to explore the effect of the substrate type on the phosphorus removal performance. In Figure 1(a)–(c) the time course of phosphate concentrations at the beginning of the anaerobic period, at the end of the aerobic and anaerobic phases during 30 days with alternating aerobic–anaerobic conditions is compared for the three different carbon sources. For the three operations, there was no obvious fluctuation in the phosphate removal; the phosphate removal performance and phosphate release during the anaerobic period increased with the increase in operation time and the final phosphate removal efficiencies under each condition were 93.8% (POSBR1), 84.7% (POSBR2) and 77.3% (POSBR3), respectively. After 24 days, POSBR1 showed substantial phosphate removal and eventually high EBPR was achieved (Figure 1(a)). POSBR2 and POSBR3 did not achieve excellent removal performance like that of POSBR1 during the operation, although the efficiency increased. Acetate as a sole carbon source enhanced the performance of phosphate removal most significantly. Moreover, the phosphate removal activities with the other two carbon sources were lower than that with acetate. These results agree with the previous reports that VFAs, such as acetate, would produce more reliable EBPR performance than other organic substrates.[26,27] A clear relationship between the carbon source and the PO_4_-P removal efficiency was observed under POSBR conditions, and significant differences in the removal efficiencies were noted as the starch concentration varied.

The number of times that the concentration of MLVSS increased compared to the initial value in three experiments is shown in Figure 1(d). Compared with the initial volume, the final microorganism concentrations were 2.16 times (POSBR1), 1.91 times (POSBR2) and 1.79 times (POSBR3) as much as those of the initial MLVSS. The net increments of MLVSS were 1.16, 0.91 and 0.79 times for the three conditions, respectively. The observed phosphate content of the biomass (mg P/mg MLVSS) decreased as the starch concentration increased for the three POSBRs, declining from 22.3% to 19.1% and 15.8%. These values were consistent with the phosphorus removal efficiencies and close to the previously reported values of 15%,[28] 17.5% [5] and 18% [29] for synthetic substrates, and consequently resulted in the biomass populations being dominated by PAOs.

### Treatment performance

**Figure 2.**
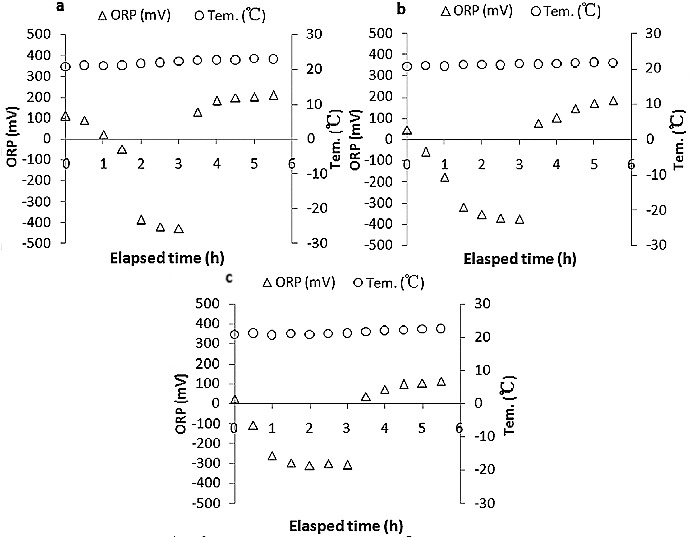
ORP and temperature of wastewater during one cycle on day 30: POSBR1 (a),[20] POSBR2 (b) and POSBR3 (c).

**Figure 3.**
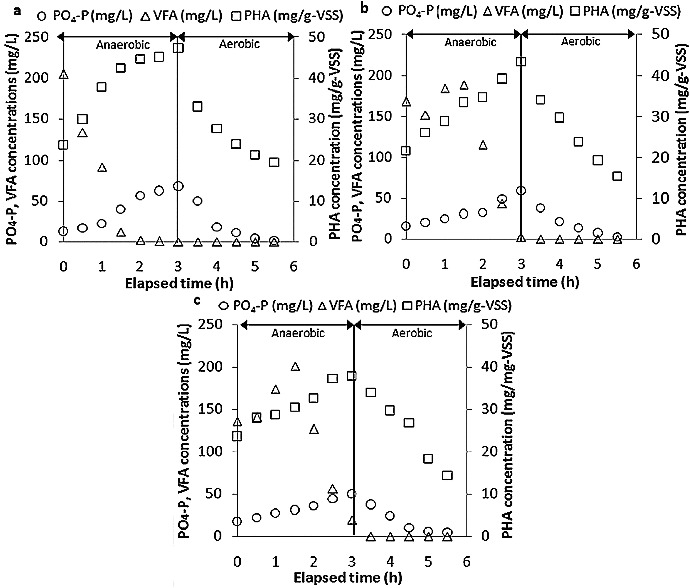
VFA, PHA and PO4-P concentrations during one cycle on day 30: POSBR1 (a),[20] POSBR2 (b) and POSBR3 (c).

The ORP and temperature of wastewater and phosphate removal performance of one cycle on day 30 are shown in Figures 2 and 3.

The biological phosphorus removal process consists of two treatment steps: biological phosphorus release and phosphorus removal. The ORP is defined in terms of the ratio of the total oxidizing power to the total reducing power of the wastewater. Figure 2 shows that the ORP readings of POSBR1 decreased rapidly in the first 3 h until a reading of about –429 mV was attained. The final ORP in the anaerobic period was decreased to −374 mV and −307 mV in POSBR2 and POSBR3, respectively. The strongly negative ORP was required during the anaerobic phase (biological phosphorus release) for the break-down of the polyphosphates and the absorption of VFAs by phosphate-accumulating bacteria. In addition, the aeration period induced the available oxygen supply and, thus, increased the DO concentration. A corresponding increase in ORP was expected, as oxygen is a driving force of ORP. The ORP of the three POSBRs approached to 200, 183 and 112 mV in the last 2.5 h, respectively, and could produce the oxidative state for phosphate uptake. The aeration was responsible for the phosphate removal taking place, and it was necessary to ensure a fairly oxidative environment. The lowest and highest ORP values were obtained in POSBR1 and there was a better reduction and oxidation environment during the whole process, which might be beneficial for PAOs accumulation and phosphate removal.

Figure 3 shows the PHA, VFA and phosphate profiles during the complete cycle in the three sets of POSBR experiments conducted with different carbon sources. Under anaerobic conditions, VFA was taken up, biomass polyphosphate was degraded and phosphate was released to the bulk solution, while PHA was synthesized in the biomass. When oxygen was present and in the absence of organic substrate, PHA was used as an energy source to take up the phosphate. Under these conditions, PAOs not only produced new biomass but also restored the storage pool of polyphosphate during the aerobic period. In POSBR1, the VFA was readily consumed during the anaerobic phase. Because of the hydrolysis and acidification of starch, the amount of VFA increased in the early period of the anaerobic stage and gradually decreased for POSBR2 and POSBR3. By comparison, the anaerobic phosphate release and PHA production decreased with increasing starch concentration in the feed. The results showed that for the PAOs, more PHA accumulated and phosphate was released in the anaerobic stage, while a higher concentration of phosphate was taken up in the aerobic period. The PHA storage was in accordance with the PO_4_-P release because the energy for PHA formation was produced by polyphosphate hydrolysis.[12] Petersen et al. [30] found that the increase of PHA in the biomass increases the phosphate uptake and subsequent polyphosphate formation. This explains the increasing PO_4_-P uptake and polyphosphate storage observed in the three POSBR operations corresponding to the PHA storage level.[20] The total amount of PHA produced from POSBR3 was minor when compared with the other two operations. Randall et al. [31] observed only marginal EBPR using starch as the carbon source. For the three different carbon source experiments in this study, the higher concentration of starch might result in low PHA accumulation and consumption, thereby limiting the amount of phosphate uptake and preventing good EBPR efficiency from being maintained.

Furthermore, the phosphate removal performance is influenced by other factors. The phosphate uptake capacity was shown to be dependent on the biomass concentration: release of phosphate increased during active growth and uptake occurred when cells reached the stationary growth phase.[32] Sidat et al. [33] reported that high biomass concentration resulted in uptake of phosphate during the entire duration of the experiment leading eventually to good phosphate removal performance, which is in accordance with our experiments. The operation under POSBR1 conditions had the highest increment of microorganism concentration compared with the other two operations. The accumulation and consumption of PHA might directly influence the phosphate removal, whereas high PHA utilization rates would stimulate the multiplication rate of PAOs. The more viable polyphosphate organisms there are in the sludge, the higher the PO_4_-P removal capacity of that sludge is. On the other hand, when assessing the overall efficiency of a biological treatment process, it is very important to take into account the temperature dependence of biological reaction rate constants.[34] Temperature affects not only the metabolic activities of the microbial population, but also the gas-transfer rates, the settling characteristics of the biological solids etc.[35] In studies performed by Panswad et al.,[1] the PAOs were found to be lower range mesophiles or perhaps psychrophiles. It was indicated that temperature could be used as a tool to control the PAOs reproduction. Optimal phosphorus release and uptake of an EBPR system were observed around 20 °C and it was beneficial for PAOs dominating among the microorganisms present.

Taken together, the results from our study showed that from a practical point of view, if the goal is to remove phosphorus from wastewater, acetate leads to the best process performance (POSBR1), with a higher yield of PHA production. For cost considerations, however, the application of starch as a carbon source sparingly with acetate, such as in POSBR2, could also achieve effective phosphate removal. Therefore, according to the results presented and the fact that ethanol is cheaper than acetate, propionate or other staple carbon sources such as starch, should be considered as an alternative carbon source if a carbon surplus in a wastewater treatment plant is needed.

## Conclusions

The obtained results demonstrated that the type of carbon source used was extremely important for phosphate removal. Most excellent phosphate removal efficiency was attained with acetate as a carbon source: highest phosphate release amount, highest PHA and polyphosphate storage and highest PAOs reproduction during the operation cycle. The carbon source fed to the microbial culture widely affects not only the PHA accumulation but also the phosphate removal performance and microorganism population. Due to the strong influence of the carbon source combination with oxygen conditions on the PHA concentration, the PHA storage mainly seemed to regulate the phosphate release and uptake. The fair EBPR performances achieved with different combinations of acetate and starch proved that using acetate plus starch as a carbon source could be considered an economically promising strategy for supporting phosphate removal from wastewaters. 

## References

[cit0001] Panswad T, Doungchai A, Anotai J (2003). Temperature effect on microbial community of enhanced biological phosphorus removal system. Water Res..

[cit0002] Jeon CO, Park JM (2000). Enhanced biological phosphorus removal in a sequencing batch reactor supplied with glucose as a sole carbon source. Water Res..

[cit0003] Mino T, van Loosdrecht MCM, Heijnen JJ (1998). Microbiology and biochemistry of the enhanced biological phosphate removal process. Water Res..

[cit0004] Yagci N, Artan N, Cokgör EU, Randall CW, Orhon D (2003). Metabolic model for acetate uptake by a mixed culture of phosphate- and glycogen-accumulating organisms under anaerobic conditions. Biotechnol Bioeng..

[cit0005] Wentzel MC, Ekama GA, Loewenthal RE, Dold PL, Marais GR (1989). Enhanced polyphosphate organism cultures in activated sludge systems. Part 2. Experimental behavior. Water SA..

[cit0006] Nelson JK, Puntenney JL (1983). Performance comparison of the air and high-purity-oxygen activated sludge systems.

[cit0007] Chen ZT, Li SG, Han GY, Xie YC (2008). Application of micro-bubble pure oxygen aeration technique to the treatment of industrial wastewater. Ind Water Treat..

[cit0008] Hesselmann RPX, Von Rummell R, Resnick SM, Hany R, Zehnder AJB (2000). Anaerobic metabolism of bacteria performingenhanced biological phosphate removal. Water Res..

[cit0009] Oehmen A, Zeng RJ, Yuan Z, Keller J (2005). Anaerobic metabolism of propionate by polyphosphate-accumulating organisms in enhanced biological phosphorus removal systems. Biotechnol Bioeng.

[cit0010] Yagci N, Artan N, Cokgör EU, Randall CW, Orhon D (2003). Metabolic model for acetate uptake by a mixed culture of phosphate- and glycogen-accumulating organisms under anaerobic conditions. Biotechnol Bioeng..

[cit0011] Oehmen A, Yuan Z, Blackall LL, Keller J (2005). Comparison of acetate and propionate uptake by polyphosphate accumulating organisms and glycogen accumulating organisms. Biotechnol Bioeng..

[cit0012] Hollender J, Van der krol D, Kornberger L, Gierden E, Dott W (2000). Effect of different carbon sources on the enhanced biological phosphorus removal in a sequencing batch reactor. World J Microb Biot..

[cit0013] Pijuan M, Casas C, Baeza JA (2009). Polyhydroxyalkanoate synthesis using different carbon sources by two enhanced biological phosphorus removal microbial communities. Process Biochem..

[cit0014] Rustrian E, Delgenes JP, Moletta R (1996). Effect of the volatile fatty acids on phosphate uptake parameters by pure cultures of Acinetobacter. Lett Appl Microbiol..

[cit0015] Satoh H, Ramey WD, Koch FA, Oldham WK, Mino T, Matsuo T (1996). Anaerobic substrate uptake by the enhanced biological phosphorus removal activated sludge treating real sewage. Water Sci Technol..

[cit0016] Puig S, Coma M, van Loosdrecht MCM, Colprim J, Balaguer MD (2007). Biological nutrient removal in a sequencing batch reactor using ethanol as carbon source. J Chem Technol Biot..

[cit0017] Kargi F, Uygur A, Başkaya HS (2005). Phosphate uptake and release rates with different carbon sources in biologicalnutrient removal using a SBR. J Environ Manage..

[cit0018] Carucci A, Lindrea K, Majone M, Ramadori R (1999b). Different mechanisms for the anaerobic storage of organic substrates and their effect on enhanced biological phosphate removal (EBPR). Water Sci Technol..

[cit0019] Usharani K, Lakshmanaperumalsamy P (2010). Bio-treatment of phosphate from synthetic wastewater using Pseudomonas sp YLW-7. J Appl Sci Environ Manage.

[cit0020] Wei J, Imai T, Higuchi T, Yamamoto K, Sekine M (2013). Effect of different anaerobic-aerobic time on the biological phosphorus removal by a sequencing batch reactor using pressurized pure oxygen. J Water Environ Technol.

[cit0021] Jeon CO, Lee DS, Lee MW, Park JM (2001). Enhanced biological phosphorus removal in an anaerobic-aerobic sequencing batch reactor – effect of pH. Water Environ Res..

[cit0022] Lopez C, Pons MN, Morgenroth E (2006). Endogenous processes during long-term starvation in activated sludge performing enhanced biological phosphorus removal. Wat Res..

[cit0023] Broughton A, Pratt S, Shilton A (2008). Enhanced biological phosphorus removal for high-strength wastewater with a low rbCOD:P ratio. Bioresour Technol..

[cit0024] American Public Health Association (1989). Standard methods for the examination of water and wastewater.

[cit0025] Shi H, Shiraishi M, Shimazu K (1997). Metabolic flux analysis for biosynthesis of poly(β-hydroxybutyric acid) in Alcaligenes eutropha from various carbon source. J Fermentation Bioeng.

[cit0026] Lin CK, Katayama Y, Hosomi M, Murakami A, Okada M (2003). The characteristics of the bacterial community structure and population dynamics for phosphorus removal in SBR activated sludge processes. Water Res..

[cit0027] Okada M, Murakami A, Lin CK, Ueno Y, Okubo T (1991). Population dynamics of bacteria for phosphorus removal in sequencing batch reactor (SBR) activated sludge processes. Water Sci Technol..

[cit0028] Crocetti GR, Hugenholtz P, Bond PL, Schuler A, Keller J, Jenkins D, Blackall LL (2000). Identification of polyphosphate accumulating organisms and design of 16S rRNA-directed probes for their detection and quantitation. Appl Environ Microbiol..

[cit0029] Appeldoorn KJ, Kortstee GJJ, Zehnder AJB (1992). Biological phosphate removal by activated sludge under defined conditions. Water Res.

[cit0030] Petersen B, Temmink H, Henze M, Isaacs S (1998). Phosphate uptake kinetics in relation to PHB under aerobic conditions. Water Res..

[cit0031] Randall AA, Benefield LD, Hill WE (1997). Induction of phosphorus removal in an enhanced biological phosphorus removal bacterial population. Water Res..

[cit0032] Momba MNB, Cloete TE (1996). The relationship of biomass to phosphate uptake by Acinetobacter junii in activated sludge mixed liquor. Water Res..

[cit0033] Sidat M, Kasan HC, Bux F (1999). Laboratory-scale investigation of biological phosphate removal from municipal wastewater. Water SA.

[cit0034] Mulkerrins D, Dobson ADW, Colleran E (2004). Parameters affecting biological phosphate removal from wastewaters. Environ Int..

[cit0035] Crites R, Tchobanoglous G (1998). Chapter 7: Biological treatment and nutrient removal. Small and decentralised wastewater management systems.

